# Phosphorylated C/EBPβ Influences a Complex Network Involving YY1 and USF2 in Lung Epithelial Cells

**DOI:** 10.1371/journal.pone.0060211

**Published:** 2013-04-01

**Authors:** Victoria Viart, Jessica Varilh, Estelle Lopez, Céline René, Mireille Claustres, Magali Taulan-Cadars

**Affiliations:** 1 UFR de Médecine, Université Montpellier1, Montpellier, France; 2 INSERM U827, Laboratoire de Génétique de Maladies Rares, Montpellier, France; 3 Laboratoire de Génétique Moléculaire, Hôpital Arnaud de Villeneuve, CHU Montpellier, Montpellier, France; University of Texas Health Science Center, United States of America

## Abstract

The promoter of the cystic fibrosis transmembrane conductance regulator gene *CFTR* is tightly controlled by regulators including CCAAT/enhancer binding proteins (C/EBPs). We previously reported that the transcription factors YY1 and USF2 affect *CFTR* expression. We can now demonstrate that C/EBPβ, a member of the CCAAT family, binds to the *CFTR* promoter and contributes to its transcriptional activity. Our data reveal that C/EBPβ cooperates with USF2 and acts antagonistically to YY1 in the control of *CFTR* expression. Interestingly, YY1, a strong repressor, fails to repress the *CFTR* activation induced by USF2 through DNA binding competition. Collectively, the data strongly suggest a model by which USF2 functionally interacts with YY1 blocking its inhibitory activity, in favour of C/EBPβ transactivation. Further investigation into the interactions between these three proteins revealed that phosphorylation of C/EBPβ influences the DNA occupancy of YY1 and favours the interaction between USF2 and YY1. This phosphorylation process has several implications in the *CFTR* transcriptional process, thus evoking an additional layer of complexity to the mechanisms influencing *CFTR* gene regulation.

## Introduction

The CCAAT/enhancer binding proteins (C/EBPs) encompass a family of structurally similar yet functionally and genetically distinct transcription factors with functional roles in a number of physiological activities [1]. In mammals there are six members including C/EBPα, C/EBPβ, C/EBPδ and C/EBPγ [2]. C/EBP expression has been linked to development, cellular differentiation and regulation of tissue specific gene expression [2], [3]. The C/EBPs are essential in a variety of tissues such as the lung where spatial and temporal regulation of *CFTR* expression is functional in maturation [3], [4]. Previous findings of several independent investigators demonstrated C/EBPδ as a positive regulator acting on the *CFTR* promoter [5], [6], [7]. In addition, Nuthall *et al*. [8] demonstrated that C/EBPβ binds to a DNase I-hypersensitive site, only present in tissue expressing *CFTR*. Through the functional analysis of sequence variations within the *CFTR* promoter, our laboratory identified other trans-acting proteins including YY1 and USF2 with repressor and activator activities, respectively [9], [10], [11]. However, the mechanistic basis of these activities has not been fully elucidated. Review of the *CFTR* gene sequence between −226 and +135 bp upstream of the major transcription initiation site [12], indicates that this region includes a C/EBPβ binding element that immediately flanks YY1 and USF binding motifs.

Through the differential usage of AUG codons within the same transcript, *C/EBPβ* encodes the different polypeptide isoforms liver-enriched activatory protein (LAP) [13] and liver-enriched inhibitory protein (LIP) [14], [15]. LAP contains both transcription activating and bZIP domains. LIP has only the bZip domain but can dimerize and bind DNA and acts as a transcriptional repressor [14].

Determining the function of C/EBP transcription factors may be achieved in part through protein-protein interactions [1]. Recent findings indicate that interaction of C/EBPs with non-C/EBP transcription factors may be relevant. Indeed Crawford *et al*. [16] reported that C/EBPγ regulates gene expression in human bronchial epithelial cells and this regulation is modified in part by interaction with YY1. In addition, C/EBPδ was reported to compete with USF for overlapping sites in the negative regulatory region of the HIV-1 long terminal repeat gene *HIV-1 LTR* [17]. C/EBPβ has also been shown to control cell-type-specific activity of viral gene through formation of a complex with YY1 [18]. The authors also reported that the YY1 activity is dependent on its functional interaction with an adjacent sequence [18].

Alternatively, multiple phosphorylation events have been shown to be critical in the regulation of members of the C/EBP family [2]. Human C/EBPβ has several known phosphorylation sites which are important for its intracellular localization and transcriptional activity [13], [19], [20], [21]. Indeed, C/EBPβ phosphorylation may lead to enhanced nuclear translocation [19], inactivation of inhibitory activities [22] or an increase in the potency of its transcriptional activation domain [20], [21].

In this report, we investigate the role of C/EBPβ in the control of *CFTR* gene expression and attempt to unravel the molecular mechanisms involved. Considering that C/EBPβ phosphorylation has already been demonstrated to modulate its DNA binding capacity and the transcriptional activities of other genes [19], we wished to evaluate the implication of this posttranslational modification in *CFTR* transcription.

## Materials and Methods

### DNA Plasmids and Constructions

For transfection assays the following expression vectors were used: pBS-C/EBPβ (S. McKnight, UT Southwestern Medical Centre), pCMV-LIP and pCMV-LAP (P. Gos, Département de Biologie Moléculaire), pCMV-Flag-A-C/EBP (V. Rishi, Laboratory of Metabolism, NCI, NIH), pCDNA3-hLAP-T235A (J. Scharwtz, Department of Physiology, University of Michigan), pCR3-USF2 (B. Viollet, Department of Endocrinology, Metabolism and Cancer) and pCDNA3-YY1 (E. Bonnefoy, Laboratoire de Régulation de la Transcription et Maladies Génétiques).

To study the importance of the C/EBPβ *cis*-acting elements, mutations within the core CAAT were generated from the WT-pGL3 plasmid using an oligonucleotide-directed mutagenesis system (QuickChange II™ Site-Directed Mutagenesis Kit, Stratagene) according to the manufacturer’s instructions. Mutagenesis primers are available on demand. The presence of mutations and the sequences fidelity were verified by dideoxynucleotide sequencing.

### Transient Transfections

Human bronchial epithelial cells, Beas2B, expressing endogenous *CFTR*, were transiently transfected as previously described [11] except for some minor modifications. They were seeded in 96-wells and transfected using Fugene6® reagent (Roche diagnostics) with 0.072 µg of reporter *CFTR* promoter plasmid, 0.008 µg of internal control pRL-SV40 containing Renilla luciferase and 0.004 to 0.08 µg of each expression vector. Transfections with siRNA were performed using Fugene6® reagent (Roche diagnostics) with 1.2 µl of either control (non-targeting siRNA: sc-37007 from Santa Cruz, Clinisciences) or C/EBPβ siRNA (sc-44251, Santa Cruz, Clinisciences) for three wells. When indicated, medium containing sodium fluoride (NAF, a Ser/thr phosphatase inhibitor) was added to cells. All luciferase activities represent at least three independent experiments with each construct tested in triplicate per experiment.

### Western Blot Analysis and Immunoprecipitation Assays

Protein extracts, resuspended directly in 1X Laemmli sample buffer, were resolved on a 10% SDS-polyacrylamide gel as previously described [9] and transferred to an Immobilon PVDF membrane (Westran® Clear Signal, Whatman). The membranes were incubated with the indicated antibodies in 5% skimmed milk overnight at 4°C: anti-C/EBPβ (sc-150X, 1∶2500 dilution), anti-USF2 (sc-862X, 1∶5000 dilution) or anti-YY1 (sc-1703X, 1∶2500 dilution) purchased from Santa Cruz, Clinisciences; anti-phospho-C/EBPβ (Thr235) (1∶1000 dilution) from Cell Signaling Technology; anti-CFTR (clone 24-1, dilution of 1∶200) from R&D Systems Europe; and anti-Flag (dilution of 1∶500) and anti-laminA/C (dilution of 1∶250) from Sigma-Aldrich Corporation. The membranes were then washed and incubated with an appropriate horseradish peroxidase (HRP) conjugated secondary antibody (sc-2004 or sc-2005, SantaCruz) at 1∶40000 in PBS-5% milk. The level of lamin A/C was assayed for internal control of protein loading. For immunoprecipitation assays, cells were resuspended in RIPA buffer (50 mM Tris/HCl, pH 7.4, 150 mM NaCl, 1% NP40, 0.5% sodium deoxycholate, 0.1% SDS and 5mM EDTA, protease inhibitors cocktail) before being immunoprecipitated using ExactaCruz F kits (sc-45043, Santa Cruz, Clinisciences). Immunoprecipitations were performed using 3 µg of specific (as indicated) or non-specific (anti-HA 12CA5, Roche Applied Science) antibodies. Extensively washed immunoprecipitates were then separated in SDS-PAGE gel and analyzed by western blotting.

### Reverse-transcriptase and Quantitative PCR

Total RNAs were extracted using the RNeasy Plus Mini kit (Qiagen SA). Reverse transcription was performed as previously described [9], and cDNA were amplified with *CFTR* primers (Table1) using the LightCycler® 480 Real-Time PCR System (Roche Applied Science) as recommended by the manufacturers. The endogenous levels of either *CFTR* mRNA or *C/EBPβ* mRNA were normalized to the expression of endogenous *GAPDH* mRNA, used as an internal control (primer listed in table 1). All PCR reactions were performed in triplicate in at least three independent experiments.

**Table 1:** Sequences of oligonucleotides and primers used.

Site directed mutagenesis experiments	
b1M	5′-gggagtcagaatcgggaatgggaggtgcgg-3′
b2M	5′-ggtgcgtagtgggaggagaaagccgct-3′
b3M	5′-aagccgctagagcatatttggggccggac-3′
b4M	5′-acccagagtagtaggtccttggcattaggagcttg-3′
b5M	5′-ttggcattaggagcatgagcccagacggc-3′
EMSA probes	
b1wt	5′aggctgggagtcagaatcgggaaagggaggtgcggggcgg-3′
b2wt	5′ggtgcgtagtgggtggagaaagccgctagagcaaatttgg-3′
b3wt	5′-ggagaaagccgctagagcaaatttggggccggaccaggca-3′
b4wt	5′-cccagagtagtaggtctttggcattaggagcttgagccca-3′
b5wt	5′-aggtctttggcattaggagcttgagcccagacggccctag-3′
b1M	5′-aggctgggagtcagaatcgggaatgggaggtgcggggcgg-3′
b2M	5′-ggtgcgtagtgggaggagaaagccgctagagcaaatttgg 3′
b3M	5′-ggagaaagccgctagagcatatttggggccggaccaggca-3′
b4M	5′-cccagagtagtaggtccttggcattaggagcttgagccca-3′
b5M	5′-aggtctttggcattaggagcatgagcccagacggccctag-3′
**Classic PCR for ChIP analyses**	
*CFTR* promoter F	5′-gaggctgggagtcagaatcgg-3′
*CFTR* promoter R	5′-catggtctctCgggcgctggggt-3′
Negative control F	5′-tgtggggagggaaatagatg-3′
Negative control R	5′-gcagagtttgcagtgagctg
qPCR ChIP analyses	
*CFTR* promoter F	5′-gagaaagccgctagagcaaa-3′
*CFTR* promoter R	5′-tcactgcccaggttaaaagc-3′
**qPCR for endogenous mRNA analyses**	
CFTR qPCR F	5′-ggaaagagaatgggatagagagc-3′
CFTR qPCR R	5′-agaacacggcttgacagctt-3′
GAPDH F	5′-ggacctgacctgccgtctagaa-3′
GAPDH R	5′-ggtgtcgctgttgaagtcagag-3′

### Formaldehyde Cross-linking and Chromatin Immunoprecipitation (ChIP)

ChIP assays were performed using the Chromatin Immunoprecipitation Assay Kit (Upstate) following Upstate ChIP protocols, with minor modifications. As indicated, Beas2B cells were transfected with either expression vectors or C/EBPβ siRNA and/or treated with 10 mM of NAF for 4 hours. Prior to harvesting, cells were incubated with 5 mM of DTBP for 30 min at RT, followed by treatment with formaldehyde 1% for 5 min. For each immunoprecipitation, 2×10^6^ cell equivalents of lysate were used. Following shearing of the crosslinked chromatin by sonication, chromatin was incubated at 4°C on a rotating stand overnight with either 3 µg of anti-C/EBPβ, anti-USF2, anti-YY1 or non-specific antibodies (anti-HA). ChIP samples (immunoprecipitated or input DNA) were analyzed by either classical PCR or quantitative PCR. For both analyses, primers used for *CFTR* promoter amplification are reported in Table 1. As a negative control, PCR analysis was carried out using primers from a region of the human *CFTR* gene which lacks both C/EBPβ and YY1 motifs: 5′-TGTGGGGAGGGAAATAGATG-3′ and 5′-GCAGAGTTTGCAGTGAGCTG-3′. The fold change in the specific binding was normalized to mock input values. For quantitative ChIP, all PCR reactions were performed in triplicate in a final volume of 10 µl containing 2 µl of sample, 5 µM of each primer (Table 1), 0.1 µl of Universal Probe ≠27 (Roche Applied Science) and 5 µl of LightCycler® 480 probes mastermix using the LightCycler® 480 Real-Time PCR System (Roche Applied Science). Experiments were repeated at least three times, averaged and expressed relative to the input signal and to the negative control (non-specific, NS, antibody).

### Nuclear Extract and Electrophoretic Mobility Shift Assays (EMSA)

Nuclear extracts were prepared from Beas2B cells as previously described [10]. Double-stranded oligonucleotides containing each wild-type or degenerated C/EBPβ motif (Table 1) identified within the minimal promoter were labelled and incubated with 10 µg of nuclear proteins in 10 µl of binding buffer (2x) containing 0.5 µg polydIdC at RT for 15 min. As indicated, purified antibodies (anti-C/EBPβ, anti-YY1 or anti-HA) were incubated with nuclear extracts before the addition of labelled probes. Purified C/EBPβ proteins were produced using TNT Quick Coupled Transcription/Translation (Promega).

### Statistical Analyses

Transfection data are expressed as the mean ± S.E. Paired comparisons were made using student’s *t*-test. Data were considered statistically significant at *p*<0.05. All graphical data and statistical analyses were generated with GraphPAD Prism software (Version 3.0). Densitometric analyses were performed using computerized densitometry and Quantity One® software (Biorad). Densitometric values for both protein band (immunoblot) and promoter *CFTR* amplicons (ChIP) were determined in areas of equal size and were reported in normalized arbitrary units relative to either expression of LaminA/C protein or input (non-*immunoprecipitated* chromatin), respectively.

## Results

### C/EBPβ Binds to and Activates the CFTR Promoter

**Figure 1:**
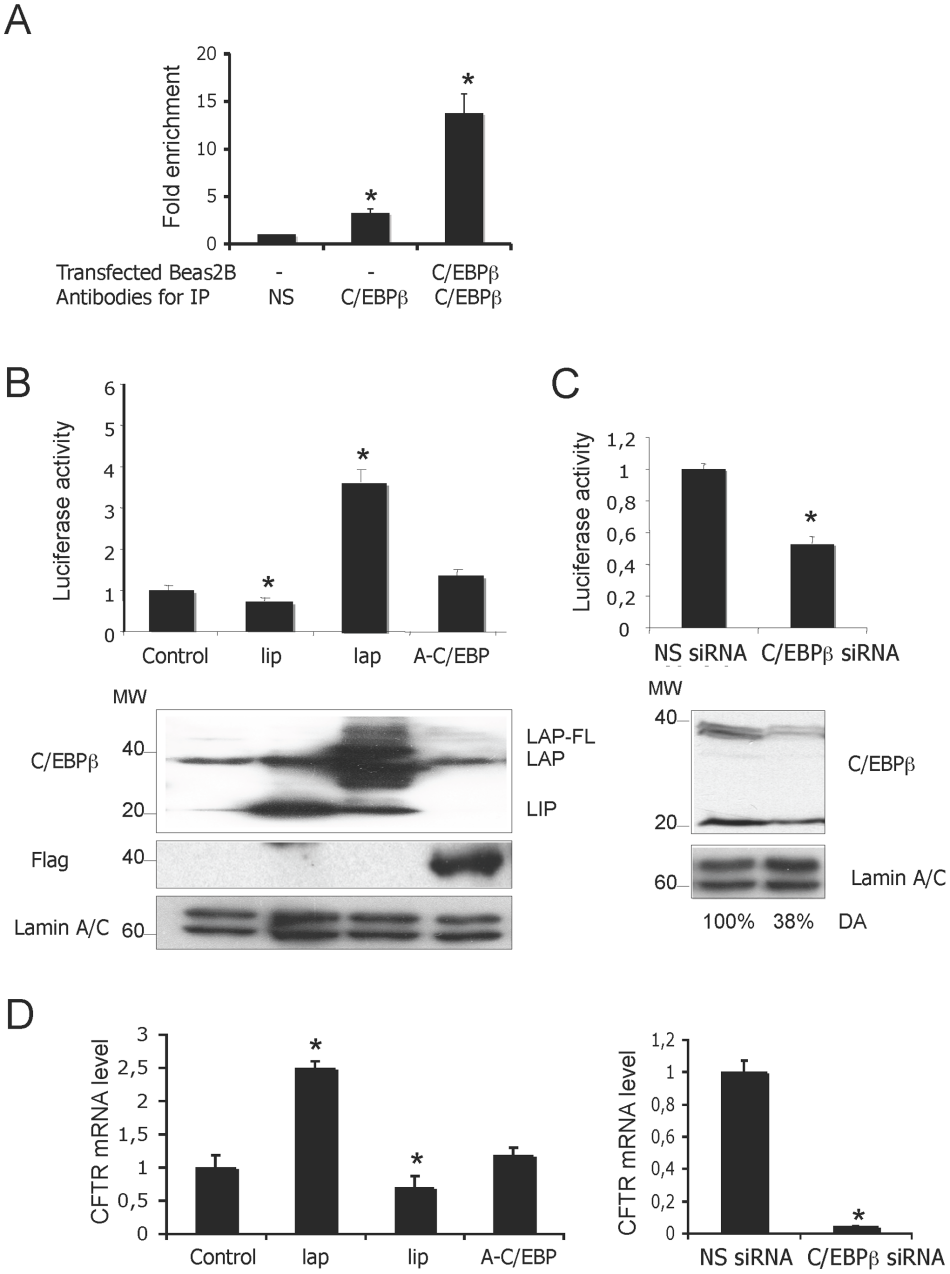
C/EBPβ activates *CFTR* promoter activity. (A) Assessment of C/EBPβ binding to the minimal *CFTR* promoter by ChIP analysis using quantitative PCR. Cells were transfected or not with C/EBPβ plasmid as indicated. Extracts were immunoprecipitated IP with either an anti-C/EBPβ or a non-specific antibody (HA also denoted NS). DNA from immunoprecipitates and input DNA (which represents 5% of total chromatin) were analyzed by quantitative PCR using primers amplifying the minimal *CFTR* promoter. Input (IN) corresponds to the amplification of total DNA and serves to normalize *CFTR* amplification as described in the Materials and Methods section. Data are expressed as fold enrichment of DNA associated with the indicated immunoprecipitated antibody relative to a 1/20 dilution of IN and specific binding was determined by subtracting binding with NS antibody. The asterisk (*) indicates that the value is statistically significant (*p*<0.05) (B) The *CFTR* promoter construct (−226 to +135) in the Luc reporter vector was co-transfected with 0.04 µg of C/EBPβ LAP or LIP, or A-CEBP expression vectors. The position of the C/EBPβ isoforms LAP (35 kDa and 38 kDa) and LIP (20 kDa) are indicated on immunoblots. Flag antibody was used for revealing A-CEBP form over-expression. (C) The *CFTR* promoter construct was co-transfected with either a non-specific (NS) siRNA or specific C/EBPβ siRNA. Densitometric Analysis (DA) was performed as described in the Materials and Methods section. (D) Effect of C/EBPβ on *CFTR* mRNA level in Beas2B epithelial cell lines. Cells were transfected with the different forms of C/EBPβ (left panel) and the C/EBPβ-specific siRNA (right panel**)**. The mRNA expression level following transfection of either an empty vector or a control siRNA was then set as 1. The asterisk reflects the statistical significance set at P<0.05.

To evaluate whether C/EBPβ binds to the minimal *CFTR* promoter, we performed chromatin immunoprecipitation (ChIP) assays using crosslinked protein-DNA complexes prepared from Beas2B cells. The immunoprecipitates obtained with antibodies directed against the C-terminus of C/EBPβ and a non-specific control protein were used as templates to amplify a 360 bp DNA fragment corresponding to the minimal *CFTR* promoter. Quantitative PCR (qPCR) amplifications of DNA immunoprecipitated by anti-C/EBPβ revealed that endogenous C/EBPβ binds to the *CFTR* promoter in Beas2B cells (Figure 1A). This binding was increased when C/EBPβ expression (Figure 1A) was enforced after normalization to input values. The control antibody was unable to precipitate any DNA target (Figure 1A). As a negative control for the C/EBPβ antibody, ChIP analysis was performed on another region of the *CFTR* gene devoid of any C/EBPβ binding motif (data not shown).

To measure the functional contribution of C/EBPβ towards promoter activity, reporter gene assays were performed with a *CFTR* promoter fused to a luciferase-coding cDNA in Beas2B cells. Overexpression of the C/EBPβ-LAP construct resulted in a 3.7 fold increase promoter activity (Figure 1B, upper panel). As expected, the truncated C/EBPβ-LIP form did not activate the *CFTR* promoter and the observed decrease in promoter activity supported a dominant negative influence of the LIP isoform as previously reported [2]. The functional role of C/EBPβ was corroborated by the almost complete abolishment of *CFTR* gene promoter activation following transient co-transfections of the dominant-negative (A-C/EBP), reported to dimerize with C/EBPβ thus blocking its DNA binding (Figure 1B, upper panel). To investigate whether the C/EBPβ transcriptional activity correlates with the transient expression level of the different isoforms in Beas2B, we performed western blot experiments. Immunoblotting with anti-C/EBPβ antibody revealed three major protein bands of 35–38 (LAP) and 20 kDa [13] (Figure 1B, lower panel). The LAP constructs can also make small amount of LIP because of internal translation initiation. To further confirm that C/EBPβ transactivates *CFTR*, a knockdown strategy was used to reduce the endogenous C/EBPβ protein level in Beas2B cells. Transient transfections of siRNA directed against C/EBPβ reduced the C/EBPβ expression by 60% (Figure 1C, lower panel) and significantly reduced the *CFTR* promoter activity (Figure 1C, upper panel), confirming the activator role of C/EBPβ. As a control, the cells were transfected with a “non-silencing” siRNA predicted to not target any gene. To ensure that the C/EBPβ influences endogenous *CFTR* mRNA, RT-qPCR assays were performed. As observed in Figure 1D, C/EBPβ induced an increase in the *CFTR* mRNA level in Beas2B cell lines; this positive effect on transcription was confirmed by using C/EBPβ-specific siRNA. Collectively, these findings firmly support an activating effect of C/EBPβ on the *CFTR* promoter.

### Mutation of a C/EBPβ Binding Motif Reduces the CFTR Transcriptional Activity

**Figure 2:**
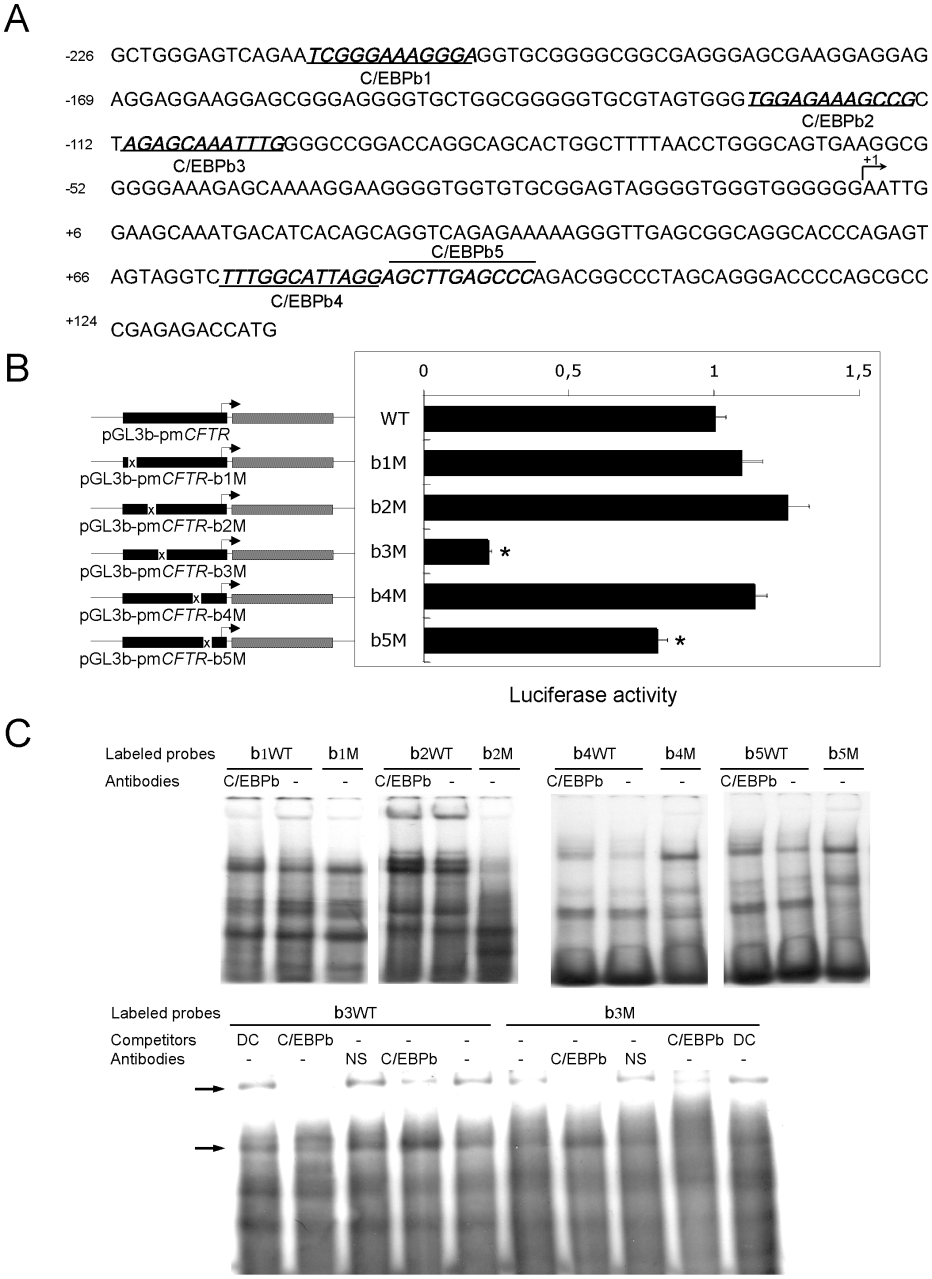
The C/EBPβ motif located at position −111/−100 is important for *CFTR* transcriptional activity. (A) Sequence of the *CFTR* minimal promoter containing five binding sites for the C/EBPβ transcription factor. The C/EBPβ motifs are underlined. The major transcriptional start site is indicated by +1. (B) Basal transcriptional activity of wild-type or degenerated C/EBPβ motifs of the *CFTR* promoter. On the left is a schematic scaled representation of the full-length pGL3 and degenerated binding motifs for the C/EBPβ transcription factor. Luciferase activity obtained with the WT-pGL3 luciferase construct was defined as 100%, and relative activities from mutant constructs are expressed as a percentage of this value. *P<0.05. (C) EMSA analysis with nuclear extracts from C/EBPβ protein-enriched Beas2B cells using wild-type or mutated labelled oligonucleotide probes (sequences listed in Table 1). Competitors (DC, Degenerated Competitors or specific competitors of C/EBPβ) or antibodies (NS, Non Specific or directed against C/EBPβ) were also used. Arrow corresponds to complexes containing C/EBPβ.

*In silico* analyses (TFsearch http://www.cbrc.jp/research/db/TFSEARCH.html, and Consite software http://asp.ii.uib.no:8090/cgi-bin/CONSITE/consite/) were performed to identify putative binding sites for the transcription factor C/EBPβ. The results indicated that the *CFTR* minimal promoter region contains different binding motifs for C/EBPβ as depicted in Figure 2A. To address whether C/EBPβ influences the *CFTR* promoter directly or exerts its effects through an indirect activation of other proteins that do directly influence *CFTR*, mutagenesis reactions of the core sequence (**T**TNNN**CAAT**NNN) of each C/EBPβ binding site in the reporter construct were performed. As shown in Figure 2B, the construction containing a nucleotide substitution (b3M) within the consensus core of the C/EBPβ motif located at the position −111/−100, named C/EBPb3, induced a significant 80% reduction in the transcriptional activity compared to the wild-type construct. In addition, the binding availability of each C/EBPβ motif was evaluated using EMSA assays. Incubation of short, radiolabeled oligonucleotide probes containing each wild-type (b1WT to b5WT) or degenerated (b1M to b5M) C/EBPβ site with nuclear extracts from Beas2B cells, resulted in different complexes that appeared altered in the mutated compared to in the wild-type context (Figure 2C). Very few complexes were formed and only with the b3WT probes. In addition the Beas2B-nuclear extracts seemed to be only slightly affected by incorporating cold C/EBP consensus sequence. Since the disruption of the C/EBPβ motif did not completely abolish *CFTR* transcription, this would suggest the implication of additional transcription factors.

### C/EBPβ Cooperates with USF2 to Activate the CFTR Promoter

**Figure 3:**
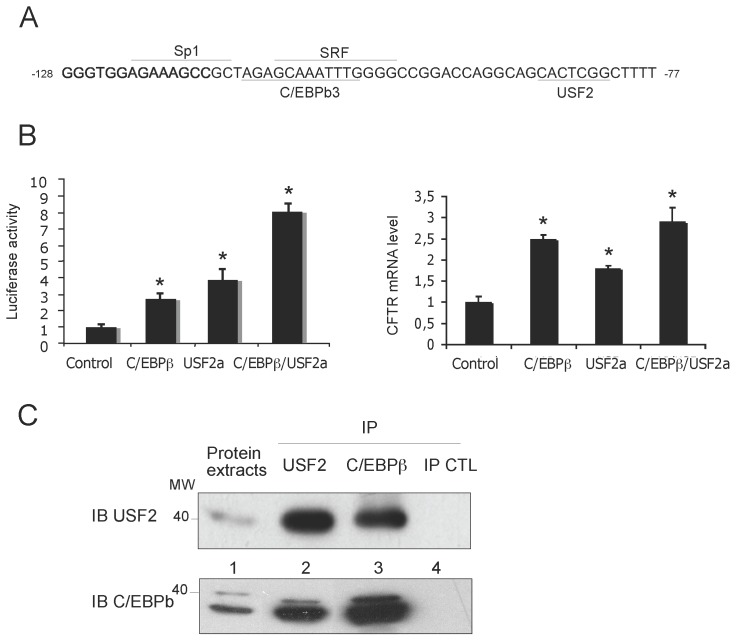
USF2 increases *CFTR* promoter activation induced by C/EBPβ. (A) Sequence of the *CFTR* minimal promoter containing binding sites for the C/EBPβ, USF2 and SRF transcription factors. The C/EBPβ motifs are underlined. (B) C/EBPβ and USF2 stimulate the *CFTR* transcriptional activity (left panel). Beas2B cells were cotransfected with the *CFTR* (0.072 µg) reporter plasmid, C/EBPβ-LAP (0.04 µg) and USF2 (0.02 µg) expression vectors as indicated. C/EBPβ and USF2 increase the endogenous *CFTR* mRNA level (right panel). Beas2B cells were cotransfected with either the C/EBPβ-LAP (0.04 µg) and USF2 (0.02 µg) expression vectors as indicated. *P<0.05. (C) Interaction between C/EBPβ and USF2 proteins. Beas2B cell extracts were immunoprecipitated with a USF2-, C/EBPβ-specific antibody (lanes 2 and 3, respectively) or an irrelevant HA antibody (lane 4). Immunoprecipitated proteins were then analyzed by western blotting using either a USF2a (upper panel) or a C/EBPβ- (lower panel) antibody. Lane 1 corresponds to whole cell extracts used for immunoprecipitation.

Based on the knowledge that C/EBPβ heterodimerization is important for its DNA binding [23], we sought to investigate the relationship between C/EBPβ and the other transcription factors that we previously characterised as binding to and regulating *CFTR* in proximity to the C/EBPb3 motif. In a first set of experiments, transient co-transfection assays were carried out to evaluate the putative cooperative role of C/EBPβ with USF2, Sp1 and SRF positive factors (binding motifs depicted on Figure 3A). The data presented in Figure 3B (left panel) demonstrate that overexpression of USF2 up-regulated the transactivation induced by the C/EBP-LAP construct in human lung cells. However, no cooperation between C/EBPβ and either SRF or Sp1 was apparent (data not shown). All results were confirmed at the mRNA level by qPCR, all be it the cooperation between C/EBPβ and USF2a was weaker than that observed by the gene reporter assays (Figure 3B, right panel). Given the functional relationship between C/EBPβ and USF proteins, we carried out co-immunoprecipitation experiments. The results of immunoblots revealed that C/EBPβ and USF2 proteins bind to each other in Beas2B cells (Figure 3C). Taken together these observations support the notion that the *CFTR* promoter is activated by the concerted action of C/EBPβ and USF transcription factors.

### Functional Antagonism between C/EBPβ and YY1 Through DNA-binding Competition

**Figure 4:**
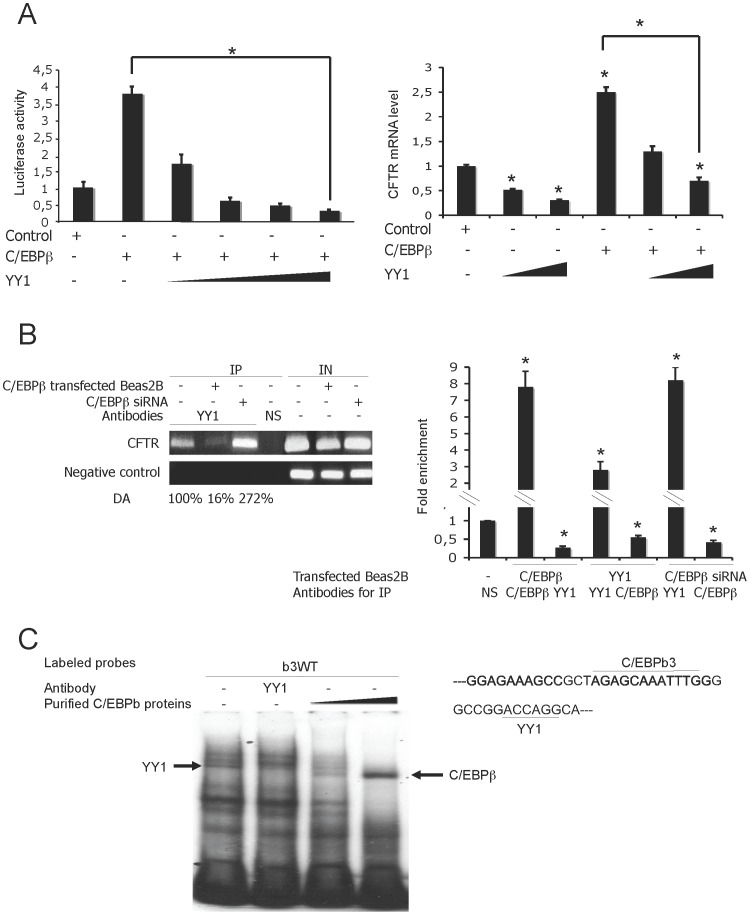
YY1 antagonizes the positive effect of C/EBP-LAP through mutually exclusive DNA binding. (A) Beas2B cells were cotransfected with a fixed amount of both *CFTR* (0.072 µg) reporter and C/EBPβ (0.04 µg) expression vectors and increasing amount of plasmid encoding YY1 (0.004 to 0.08 µg) as indicated (left panel). Endogenous *CFTR* mRNA level following C/EBPβ (0.04 µg) expression vectors and increasing amounts of plasmid encoding YY1 (0.004 and 0.08 µg) (right panel). *P<0.05. (B) Left panel: ChIP experiment was performed on cells transfected or not with either C/EBPβ plasmid or respective siRNA. Protein extracts were immunoprecipitated IP with the indicated antibody. Input (IN) corresponds to total lysate used as a control for PCR amplification of total DNA. CFTR, represents *CFTR* promoter amplification and negative control, ChIP analysis of *CFTR* sequence which lacks the YY1 binding motif. Right panel: DNA from immunoprecipitates and input DNA (which represents 5% of total chromatin) were analyzed by quantitative PCR using primers amplifying the minimal *CFTR* promoter. Data are defined as fold enrichment of DNA associated with indicated immunoprecipitated antibody relative to input chromatin and specific binding was expressed as a function of non-sepcific (NS) antibody binding set as 1. (C) Functional interplay between C/EBPβ and YY1. Mutually exclusive DNA-binding activity of YY1 and C/EBPβ at the C/EBPb3 binding site. The labelled b3WT probe was incubated with C/EBPβ-transfected Beas2B nuclear extracts in the presence of increasing amounts of purified C/EBPβ. Arrows indicate the position of C/EBPβ and YY1.

Since YY1 has been previously shown to negatively regulate the *CFTR* promoter [9], it was logical therefore to presume that C/EBPβ and YY1 be putative functional competitors for *CFTR* promoter activity. This hypothesis was investigated by transient co-transfections with both C/EBPβ and YY1 expression vectors. As observed in Figure 4A (left panel), the stimulating effect of C/EBPβ progressively decreased on addition of increasing quantities of YY1 plasmid. To determine the potential displacement of YY1 binding by C/EBPβ binding, ChIP experiments were conducted using extracts prepared from Beas2B cells transfected with either C/EBPβ expression vector or its respective siRNA. As a negative control for YY1 antibody, ChIP analysis from another region of the *CFTR* gene devoid of any YY1 motif was performed (denoted negative control). C/EBPβ overexpression significantly reduced the YY1 DNA binding activity (Figure 4B, left panel). Interestingly, the binding of YY1 to its cognate site was restored by transfection of the C/EBPβ siRNA. ChIP-qPCR confirmed that overexpression of C/EBPβ significantly decreases the YY1 binding on the *CFTR* minimal promoter (Figure 4B, right panel). Both YY1 and C/EBPβ-specific siRNA overexpression also indicated the antagonising effects of C/EBPβ and YY1. To determine whether the interaction of these two factors with their target DNA containing the C/EBPb3 motif and a YY1 binding site (Figure 4C, right panel) was either mutually inclusive or exclusive, EMSAs were conducted. As shown in Figure 4C (left panel), the YY1-binding complex was altered by increasing the C/EBPβ binding activity, suggesting their mutually exclusive binding. These results demonstrate a functional antagonism between C/EBPβ and YY1 through DNA-binding competition.

### C/EBPβ Transactivation is in Part Due to Interaction between USF2 and YY1

**Figure 5:**
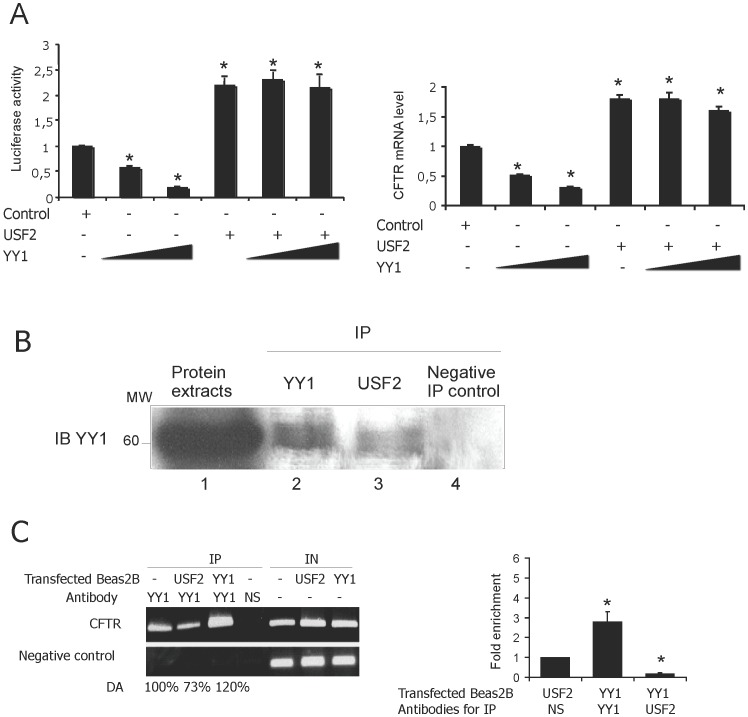
USF2 interacts with YY1 and blocks its repressive activity. (A) Left panel: Beas2B cells were cotransfected with a fixed amount of both *CFTR* (0.072 µg) reporter plasmid and USF2 (0.02 µg) expression vectors and increasing amounts of plasmid encoding YY1 (0.004 and 0.08 µg) as indicated. *P<0.05. Right panel: Endogenous *CFTR* mRNA level following transfection of USF2 (0.02 µg) expression vectors and increasing amounts of plasmid encoding YY1 (0.004 and 0.08 µg). (B) Interaction between USF2 and YY1 proteins. Beas2B cell extracts were immunoprecipitated with either specific antibodies (lanes 2 and 3) or the HA irrelevant antibody (lane 4), as indicated. Immunoprecipitated proteins were then analyzed by western blotting using a YY1-specific antibody. Lane 1 corresponds to whole cell extracts used for immunoprecipitation. (C) Left panel: ChIP analysis was performed to evaluate the YY1 DNA occupancy. Protein extracts from cells transfected or not with indicated antibodies, were immunoprecipated IP with either a specific or an irrelevant antibody (HA). Input (IN), corresponds to amplification of total DNA. CFTR, represents *CFTR* promoter amplification and negative control, ChIP analysis of the *CFTR* sequence lacking the YY1 binding motif. Right panel: DNA from immunoprecipitates and input DNA were analyzed by quantitative PCR. Data are defined as fold enrichment relative to input chromatin and specific binding was expressed as a function of non-sepcific (NS) antibody binding set as 1.

Considering the observed cooperation between C/EBPβ and USF2 and the antagonism between C/EBPβ and YY1, we wondered whether YY1 had any effect on the cooperativity between C/EBPβ and USF2. We firstly tested whether enforced YY1 expression antagonizes USF2 transactivation. Surprisingly, YY1 overexpression had no considerable effect on the *CFTR* promoter transactivation induced by USF overexpression (Figure 5A, left panel). To evaluate the effect of a combined YY1 and USF2 overexpression on the endogenous *CFTR* mRNA level, RT-qPCR assays were conducted. The results confirm the data obtained by gene reporter assays (Figure 5A, right panel). To explain the functional cooperation between C/EBPβ and USF2, we next explored whether USF might block the action of YY1 through USF2/YY1 interaction. Co-immunoprecitation experiments revealed a physical interaction between YY1 and USF2 in Beas2B cells (Figure 5B). We then tested whether enforced USF2 expression might decrease the YY1 recruitment onto the *CFTR* promoter. As presented in Figure 5C (left panel) USF2 overexpression reduced YY1 binding to the *CFTR* promoter, as shown by ChIP analyses. To confirm these data, Chip-qPCR assays were also conducted showing that USF2 overexpression significantly decreased the YY1-binding capacity by 50% (Figure 5C, right panel). Taken together, these data strongly suggest that USF2 and YY1 interact *in vivo* and this interaction seems to prevent the YY1 DNA occupancy.

### Phosphorylation of C/EBPβ is Associated with CFTR Transactivation

**Figure 6:**
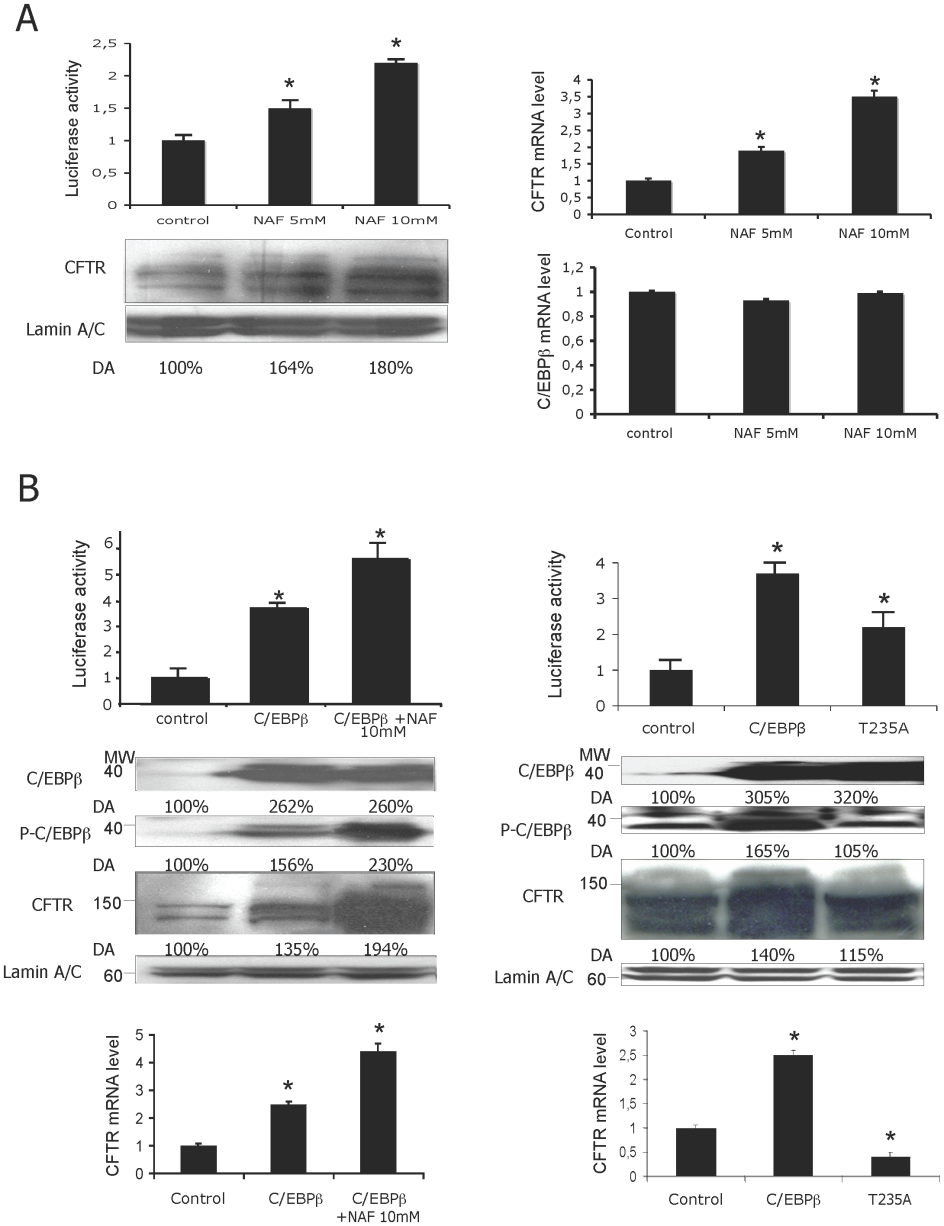
NAF treatment stimulates the *CFTR* activity induced by C/EBPβ. (A) Left panel: When indicated, Beas2B cells were incubated in the presence of NAF (left panel) at the indicated concentrations. Immunoblots showing either CFTR or LaminA/C expression are represented below the graph. Right panel: Endogenous mRNA level of either *CFTR* or *C/EBPβ* following NAF incorporation at indicated concentrations. (B) Upper panel: Combinatorial effect of C/EBPβ and NAF treatment. Cells were transfected either with C/EBPβ plasmid after NAF treatment (left panel) or with LAPT235A expression vector (right panel). Middle panel: Representative immunoblots are shown below the graphs. Lower panel: Endogenous *CFTR* mRNA level. *P<0.05.

Since the acquisition of transcriptional activity for various transcription factors including C/EBPβ requires their phosphorylation [24], we first wondered whether treatment with phosphatase inhibitors might influence the *CFTR* activity. We evaluated the effect of either sodium fluoride (NAF, a serine/threonine inhibitor) or vanadate (Vana, a tyrosine phosphatase inhibitor) treatment at different concentrations on the *CFTR* transcription. While vanadate did not modify the *CFTR* expression level (data not shown), treatment with either 5 mM or 10 mM NAF significantly increased the luciferase activity to over 1.5- and 2.5-fold, respectively (Figure 6A, left panel). Western blotting experiments using an anti-CFTR antibody demonstrated that endogenous CFTR expression correlated well with the observed luciferase activity. Densitometric analysis (denoted DA) was performed to evaluate the protein expression level and the values are indicated under the immunoblots. RT-qPCR assays confirmed the results showing an increase of 2- or 3-fold in the *CFTR* mRNA level following 5 mM or 10 mM NAF treatment, respectively (Figure 6A, right panel). RT-qPCR assays were also performed to assess the effect of NAF treatment on the C/EBPβ transcripts. The results show that incubation of either 5 mM or 10 mM NAF did not affect the endogenous C/EBPβ mRNA level (Figure 6A, right panel).

We next examined the role of phosphatase inhibitor on the C/EBPβ-mediated *CFTR* transactivation. As observed in Figure 5B, at 10 mM NAF significantly promoted *CFTR* activation induced by over-expression of C/EBPβ in Beas2B cells. In contrast, NAF treatment did not modify USF2 transactivation on the *CFTR* promoter (data not shown). Western blot assays were carried out to assess the protein expression levels under the indicated treatments. Immunoblotting with a specific anti-phospho-C/EBPβ antibody showed that treatment with NAF increased the level of phosphorylated C/EBPβ (Figure 6B, upper panel, left panel). Interestingly, immunoblotting also revealed that the level of endogenous CFTR protein increased after C/EBPβ overexpression, reaching its maximum level upon NAF incubation (Figure 6B, middle and left panel). These data were validated by using dominant negative form of C/EBP (A-C/EBP) showing that no *CFTR* expression was observed after A-C/EBP overexpression following NAF treatment (data not shown). These results indicate that NAF-induced C/EBPβ phosphorylation is involved in *CFTR* activation. To assess whether the increased *CFTR* expression could be an indirect consequence of the general effects of NAF on promoter efficiency, we performed transfection assays using mutant hLAPT235A vector (denoted T235A), in which the conserved Thr235 is mutated to Ala. As presented in Figure 6B (upper and right panel), the overexpression of the LAP expression vector containing a mutation of the Thr235 induced only a two-fold increase in the luciferase transcription. Although western blot assays revealed an increase in C/EBPβ expression level after either C/EBPβ or T235A overexpression, the anti-phospho-C/EBPβ antibody was unable to detect mutated T235A highlighting the specificity of the antibody (Figure 6B, middle and right panel). RT-qPCR assays were then performed to evaluate the effect of C/EBPβ phosphorylation on the mRNA level of endogenous *CFTR*. The results confirmed the data obtained by gene reporter assays (Figure 6B, lower panels). Interestingly, overexpression of hLAPT235A vector induced a decrease in *CFTR* mRNA level. These results indicate for the first time that C/EBPβ phosphorylation is involved in *CFTR* transcriptional activation.

### Phosphorylation Affects Both C/EBPβ and YY1 DNA Binding Abilities and Favours YY1/USF2 Interaction

**Figure 7:**
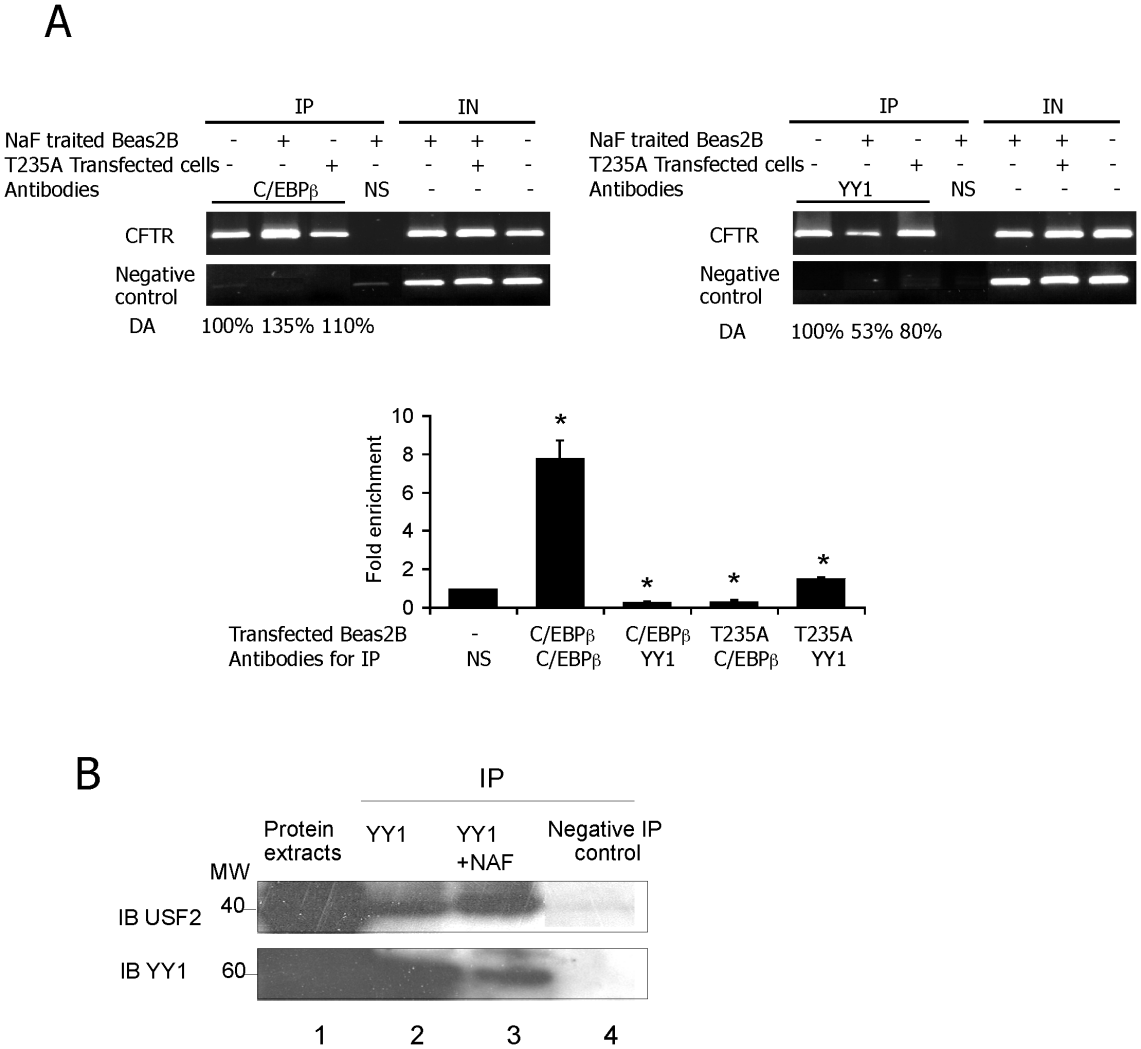
Phosphorylation of C/EBPβ affects the YY1 DNA occupancy and favours YY1/USF2 interaction. (A) Left panel: ChIP experiments were performed using NAF-treated Beas2B cells when indicated. Protein extracts were immunoprecipitated IP with the indicated antibodies. Input (IN), corresponds to total lysate used as a control for PCR amplification of total DNA and served to normalize *CFTR* amplification as described in Materials and Methods Section. CFTR, represents *CFTR* promoter amplification and negative control, ChIP analysis of *CFTR* sequence which lacks both C/EBPβ and YY1 binding motifs. Right panel: DNA from immunoprecipitates and input DNA were analyzed by quantitative PCR. Data are defined as fold enrichment relative to input chromatin and specific binding was expressed as a function of non-specific (NS) antibody (anti-HA) binding set as 1. (B) Beas2B cells were treated when indicated with NAF and protein extracts were immunoprecipitated with either an anti-YY1 (lanes 2 and 3) or an irrelevant antibody (lane 4). Immunoprecipitated proteins were then analyzed by western blotting using either an anti-USF2a or an anti-YY1 antibody. Lane 1 corresponds to whole cell extracts used for IP.

Considering that C/EBPβ appears to undergo phosphorylation in correlation with the acquisition of DNA-binding activity [2], we used ChIP assays to address the C/EBPβ activity in terms of its phosphorylation status. NAF induced-phosphorylated C/EBPβ was associated with both an increased C/EBPβ DNA binding activity and a decrease in YY1 DNA binding *in vivo* (Figure 7A, left and right panels, respectively). To validate these results, we evaluated the C/EBPβ (Figure 7A, left panel) and YY1 (Figure 7A, right panel) occupancies on the *CFTR* target sequence after overexpression of the LAPT235A mutant (denoted T235A). As depicted in Figure 7A (upper panels), LAPT235A could bind to the *CFTR* promoter but the level of binding was much lower by comparison with the phosphorylated C/EBPβ. These results were confirmed by ChIP-qPCR showing that LAPT235A overexpression induced a considerable decrease in C/EBPβ binding (nearly 3-fold) that was associated with a modest increase in YY1 binding activity (1.5-fold) when compared to endogenous DNA binding activity for either the C/EBPβ or YY1 transcription factors (set as 1), respectively (Figure7A, lower panel). The DNA binding activity for USF2 was not altered following C/EBPβ or LAPT235A overexpression (data not shown). These data show that phosphorylation of C/EBPβ increases its DNA binding ability and disrupts the YY1 occupancy of its DNA cognition site.

Consequently, we investigated whether this latter effect is mediated by interactions between YY1 and USF2. Co-immunoprecipitation assays demonstrated that NAF treatment does indeed favour the interaction between YY1 and USF2 (Figure 7B). As a positive control, immunoblotting was performed using an anti-YY1 antibody. The data presented above suggest that phosphorylation plays an important role in C/EBPβ binding to DNA. These findings also demonstrate that the phosphorylation status also influences the DNA binding properties of the YY1 factor and increases the USF2/YY1 interaction.

## Discussion

C/EBPβ and both related polypetides LAP (liver-enriched activatory protein) and LIP (liver-enriched inhibitory protein) belong to the CCAAT/Enhancer binding proteins (C/EBP) family of transcription factors. These nuclear *trans*-acting factors play pivotal roles in airway epithelial cell differentiation during lung organogenesis [25] and are involved in the expression of lung-specific and developmentally regulated genes including *SP-A* and *CFTR* [5], [6], [7], [8], [26], [27]. We recently identified other *trans*-acting factors including SRF, YY1, USF2, Sp1 and Nrf2 that contribute to *CFTR* transcriptional activity [9], [10], [11], [28]. In addition, C/EBPβ has been previously reported to be an important accessory factor for transactivation by several other transcription factors [29]. In an effort to enhance our understanding of the mechanisms that modulate the complex *CFTR* expression pattern, we investigated the functional role of C/EBPβ alongside some previously reported transcription factors.

The data presented in this report show that C/EBPβ positively influences *CFTR* promoter activity in bronchial epithelial cells. Based on knowledge of the *CFTR* promoter being quite weak, this *trans*-acting factor may be considered as a strong activator. The results obtained with the C/EBPβ-specific siRNA and with mutants for the C/EBPβ binding site demonstrate the importance of C/EBPb3 and its binding motif located within a region previously documented to contain two mutations identified in CF patients [11], [10]. The fact that the *CFTR* mRNA level was never completely abolished in our assessments is evidence of the C/EBPβ transcription factor controlling *CFTR* transcription in a coordinated fashion with other partners. We have illustrated here its cooperation with USF2. Moreover, considering the repressor role of YY1 in *CFTR* transcriptional regulation [9], we also investigated the putative competition between YY1 and C/EBPβ functional activities by transient co-transfection experiments. We demonstrated that the *CFTR* transcriptional activation elicited by C/EBPβ is antagonized by YY1 overexpression. We also showed that this functional antagonism between YY1 and C/EBPβ might be partially due to mutually exclusive DNA-binding activities. In addition, although we previously reported that USF cooperates with Sp1 to transactivate the *CFTR* gene and that YY1 antagonizes SRF, we failed to demonstrate any cooperativity between C/EBPβ and either SRF or Sp1 in our promoter context. Indeed, USFs and Sp1 may act as negative regulators when they interact with transcription factors bound simultaneously to the same site or at other sites, suggesting a dual mode of regulation of genes by these factors (28,29). Interestingly, the results of this study confirm the complex link between the multiple transcription factors that regulate *CFTR* transcription.

To further explore the relationship between the C/EBPβ, USF2 and YY1 factors, transient co-transfections were performed. Enforced expression of YY1, a powerful repressor, was unable to inhibit *CFTR* activation induced by USF2. Several concordant results strongly argue in favour of USF contributing towards C/EBPβ-mediated *CFTR* activation through a functional USF/YY1 interaction. This interaction could at least partially explain the cooperation between C/EBPβ and USF2 and the DNA competition observed between C/EBPβ and YY1. Based on these data, we propose a model of interaction between USF and YY1 by which USF decreases the inhibitory activity of YY1, either through interacting with the domain mediating the YY1 repressive activity or by impeding access of co-regulators. Indeed, p300 which has been shown to bind YY1, may be essential for YY1 repressor activity [30]. Similar mechanisms have previously been reported for other genes [18], [31]. For instance, USF2 was shown to disturb transcriptional activation by inhibiting the binding of hypoxia-inducible factor-1 to the plasminogen activator inhibitor-1 gene in hepatocytes [31]. Another study reported that C/EBPβ may alter the DNA binding of activators such as AP-1, by competition and/or by negatively regulating their transcriptional activity *via* physical interaction [18]. Thus, more extensive studies including deletions in the protein/protein interaction motifs for USF2 and YY1 factors are envisaged.

**Figure 8:**
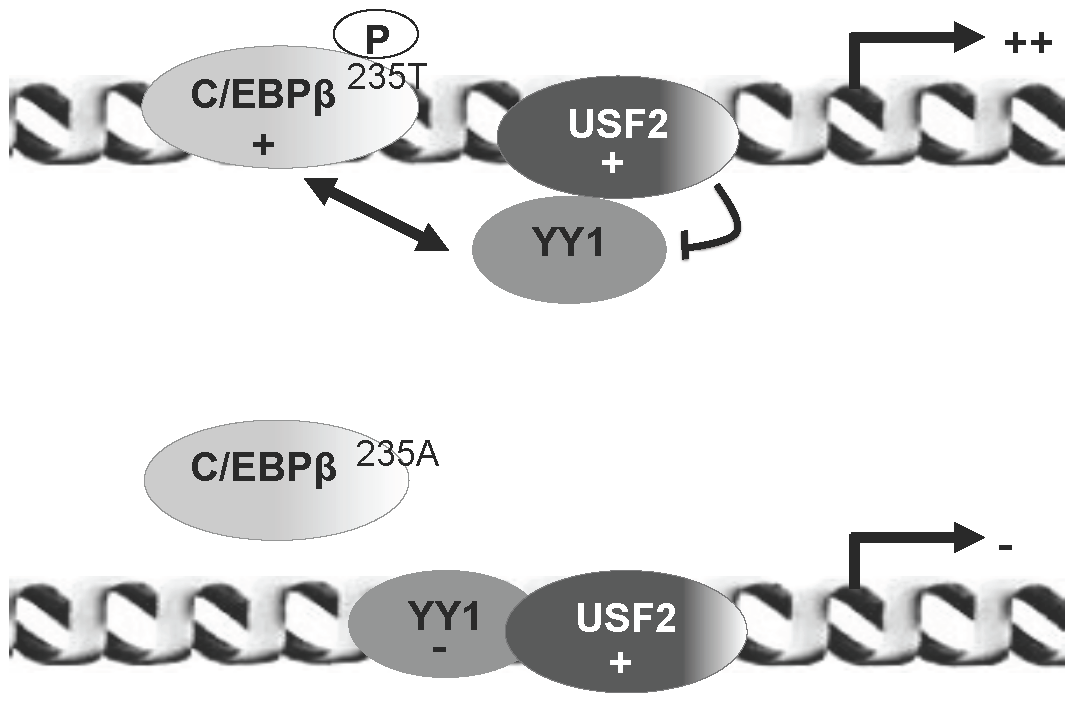
Schematic model depicting the potential mechanism that might contribute to regulation of the *CFTR* gene. In this model, coloured bubbles correspond to the transcription factors characterized in this study. Double arrow shows the functional antagonism between C/EBPβ and YY1. The upper representation corresponds to the transcriptional activation of the *CFTR* gene when C/EBPβ is phosphorylated and the lower representation corresponds to the decrease of the transcription following overexpression of a C/EBPβ form not phosphorylatable on its 235T residue.

In addition to their acquisition of transcriptional activity through selective interactions, transcription factors also require posttranslational modifications in order to function. This highlights the need to study other levels of regulation. Interestingly, it has been shown that C/EBPβ is mainly activated by serine/threonine phosphorylation, which results in its heterodimerization and an increase in its DNA binding [2]. In light of this finding, we assessed whether phosphorylation of C/EBPβ has the ability to modulate the *CFTR* transcriptional activity. By using the Ser/thr phosphatase inhibitor NAF, we demonstrated an increase in C/EBPβ phosphorylation, DNA occupancy and endogenous *CFTR* expression in bronchial epithelial cells. To confirm these results, we performed transient transfection, RT-qPCR and qChIP assays by using a LAPT235A expression vector, mutated on threonine 235. We found that this mutant protein, unable to be phosphorylated on its Thr residue, could only induce a modest activation of *CFTR* transcription compared to the increased *CFTR* expression induced by C/EBPβ overexpression and significantly altered the *CFTR* mRNA level. In addition, the overexpression of the LAPT235A protein induced a considerable decrease in the C/EBPβ DNA binding activity (observed by qChIP analysis) and an increase in the YY1 DNA occupancy. The ChIP analyses were consistent with the results obtained with the NAF treatment. However, other sites of phosphorylation, not studied in our system, might be implicated in C/EBPβ activity as was previously reported (13). These data are in agreement with a recent study showing that phosphorylation of the CREB transcription factor influences *CFTR* expression [32]. In addition, our findings are particularly interesting since only a low level of CFTR protein is sufficient to allow its normal function [33]. In this manuscript, we also demonstrate that NAF reduces the YY1 DNA binding properties and increases the interaction between YY1 and USF2. These findings strengthen a model by which specific interaction between USF2 and YY1 may result in an increase of both C/EBPβ DNA occupancy and *CFTR* transcriptional activation (Figure 8). This fine balance between negative and positive regulators such as that demonstrated here with the tissue-specific C/EBP factor might explain the finely *CFTR* regulated expression observed during the development of certain tissues including lung formation [34].

A crucial point raised is which factors help determine between antagonism and cooperation within a given cell? The answer to this question is very complex and to date not yet elucidated. One important consideration is the expression level of each transcription factor which depends on the specific cell type. For instance, it has been reported that the LIP/LAP ratio may dictate the C/EBPβ transcriptional activity and depend on the cell type [14]. In addition, while most proteins including YY1 and USF are ubiquitously expressed [35], [36], we must keep in mind that enforced expression of transcription factors may not reflect their real endogenous activities. To gain insight into how such a coordination of gene expression might occur, it will be also instructive to examine posttranslational modifications. Indeed, mounting evidence suggests that acetylation and phosphorylation of nuclear factors may be interdependent [37]. For instance, C/EBPβ recruits p300, triggers massive phosphorylation within its C-terminal domain and thereby modulates p300 activity as a co-activator [38]. Recently, other investigators revealed a cooperation between acetylation and phosphorylation in the control of GATA-1 transcription factor activity [39]. In this regard, an interesting point deserving further attention is the evaluation of both C/EBPβ phosphorylation and acetylation status including mutations of the target sites and their impact on C/EBPβ DNA binding and protein-protein interaction abilities.

This work has highlighted that such a mode of regulation involving cooperative and antagonistic interaction coupled with posttranslational modification, is an interesting avenue that we will pursue in the future to further unravel the complex regulation of the *CFTR* gene.
